# Molecular Mechanisms of AMPA Receptor Trafficking in the Nervous System

**DOI:** 10.3390/ijms25010111

**Published:** 2023-12-21

**Authors:** Yi-Yang Cao, Ling-Ling Wu, Xiao-Nan Li, Yu-Lian Yuan, Wan-Wei Zhao, Jing-Xuan Qi, Xu-Yu Zhao, Natalie Ward, Jiao Wang

**Affiliations:** 1 Laboratory of Molecular Neural Biology, School of Life Sciences, Shanghai University, Shanghai 200444, China; 15239050331@shu.edu.cn (Y.-Y.C.); rywqwxwx@163.com (X.-N.L.); m13595139460@163.com (Y.-L.Y.); vvzhao0629@shu.edu.cn (W.-W.Z.); qijingxuan@outlook.com (J.-X.Q.); zxy_9928@shu.edu.cn (X.-Y.Z.); 2 School of Medicine, Shanghai University, Shanghai 200444, China; 19990510wll@shu.edu.cn; 3 Medical Laboratory, Exceptional Community Hospital, 19060 N John Wayne Pkwy, Maricopa, AZ 85139, USA; natt.a.ward@gmail.com

**Keywords:** AMPAR, molecular mechanism, glutamate receptors, synaptic plasticity, AMPAR trafficking

## Abstract

Synaptic plasticity enhances or reduces connections between neurons, affecting learning and memory. Postsynaptic AMPARs mediate greater than 90% of the rapid excitatory synaptic transmission in glutamatergic neurons. The number and subunit composition of AMPARs are fundamental to synaptic plasticity and the formation of entire neural networks. Accordingly, the insertion and functionalization of AMPARs at the postsynaptic membrane have become a core issue related to neural circuit formation and information processing in the central nervous system. In this review, we summarize current knowledge regarding the related mechanisms of AMPAR expression and trafficking. The proteins related to AMPAR trafficking are discussed in detail, including vesicle-related proteins, cytoskeletal proteins, synaptic proteins, and protein kinases. Furthermore, significant emphasis was placed on the pivotal role of the actin cytoskeleton, which spans throughout the entire transport process in AMPAR transport, indicating that the actin cytoskeleton may serve as a fundamental basis for AMPAR trafficking. Additionally, we summarize the proteases involved in AMPAR post-translational modifications. Moreover, we provide an overview of AMPAR transport and localization to the postsynaptic membrane. Understanding the assembly, trafficking, and dynamic synaptic expression mechanisms of AMPAR may provide valuable insights into the cognitive decline associated with neurodegenerative diseases.

## 1. Introduction

The proper functioning of the brain depends on the stable transmission of information between neurons via synapses, which include excitatory and inhibitory synapses. The α-amino-3-hydroxy-5-methyl-4-isoxazoliopropionate receptor (AMPAR) and N-methyl-D-aspartate receptor (NMDAR) are two ionotropic glutamate receptors commonly involved in excitatory synaptic activity, playing a vital role in the formation of neurons and synaptic plasticity in the brain [1]. During synaptogenesis, the modulation of subunit composition and the relative abundance of AMPARs and NMDARs are considered critical in establishing functionally mature synapses.

Numerous studies have shown that the precise regulation of the isotype and the number of AMPARs on the postsynaptic membrane are key to synaptic transmission and synaptic plasticity, which help with the formation of precise neural circuits [2,3,4]. Defects in AMPAR aggregation, transport, and expression are fundamental causes of neurodegenerative disease and neurodevelopmental disorders such as Alzheimer’s disease (AD), schizophrenia (SCZ), depression, and autism.

AMPARs are tetramers formed from combinations of four subunits with different stoichiometries that possess different channel characteristics and are encoded by four different genes, *Gria1–4*. AMPARs in the adult hippocampus are generally composed of GluA1/2 or GluA2/3 [5,6]. These four subunits are highly homologous and differ mostly in the C-terminal domain, which determines their binding to different proteins during AMPAR trafficking. AMPAR trafficking processes that rely on different specific bindings of PDZ domains enable different protein-binding effects to occur in the next step. For example, the C-terminal domain of GluA1 contains type I PDZ ligands and interacts directly with SAP97, while that of GluA2 contains type II PDZ ligands and interacts directly with PICK1 and GRIP1 [7].

**Figure 1:**
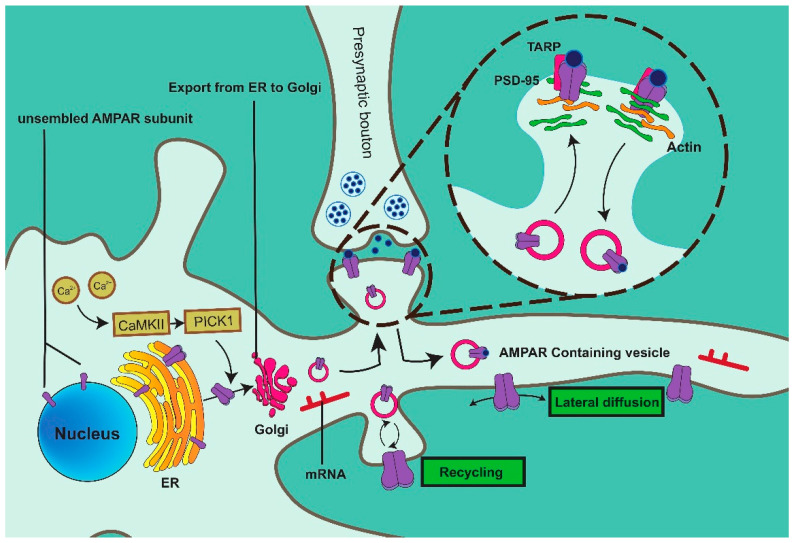
Schematic illustration of the AMPAR trafficking mechanism.

AMPARs are synthesized in the endoplasmic reticulum (ER), subsequently traveling along the actin filament within vesicles to the exocytosis site. AMPARs are released and inserted into the membrane via a fusion process (Figure 1). The expression of AMPARs on the synaptic membrane is a highly dynamic process since AMPARs are constantly circulating between the cytoplasm and the plasma membrane.

The synaptic strength of excitatory synapses is mainly regulated by the expression and activity of AMPARs, and AMPAR trafficking is a continuous recycling process. Ca^2+^ phosphorylates and activates Ca^2+^/calmodulin-dependent regulated kinase II (CaMKII), promoting the formation of the CaMKII-PICK1 complex. PICK1 binds to the C-terminal domain of GluA2 via its PDZ domain and stimulates the transportation of GluA2 to the ER membrane. AMPARs travel through the cytoplasm in the form of vesicles after being modified by the Golgi apparatus (GA). A proportion of AMPARs is directly inserted into the synaptic membrane, while other AMPARs first act at a non-synaptic site via exocytosis and subsequently move to the synapse via lateral diffusion. AMPAR-containing vesicles are transported along actin filaments to the postsynaptic density (PSD). Transmembrane AMPAR-related proteins (TARPs) preferentially bind AMPAR tetramers and subsequently bind PSD-95 through its cytoplasmic tail (CT), a mechanism that promotes AMPAR recruitment and localization to synapses, ensuring that AMPARs function in the correct position.

Long-term potentiation (LTP) and long-term depression (LTD) are the two major forms of long-lasting synaptic plasticity in mammalian neurons. Most studies on the trafficking mechanism of AMPARs have concluded that LTP and LTD depend on the exocytosis and endocytosis of AMPARs [4,8,9]. Exocytosis of AMPARs is increased during LTP induction, while enhanced endocytosis can lead to LTD [4]. Sufficient evidence indicates that LTP and LTD are the two main molecular mechanisms involved in learning, memory, and cognition, which are impaired by the disruption of AMPAR function or trafficking [10]. However, it remains uncertain whether AMPAR proliferation and recruitment are mandatory steps for all synaptic LTPs. It has been found that the pathogenesis of diseases presenting with symptoms of cognitive dysfunction, such as AD [11], intellectual disability/intellectual developmental disorder (ID/IDD) [12], autism spectrum disorder (ASD) [12,13,14], schizophrenia (SCZ) [14], and epilepsy [12], has a certain correlation with the expression and trafficking of AMPARs.

Extracellular aggregation of amyloid-β (Aβ) is a key event in the pathogenesis of AD, and the regulation of extracellular Aβ levels directly affects the onset of the disease. Using quantitative autoradiography techniques, it has been demonstrated that glutamate metabolic binding and AMPA binding are significantly reduced in the CA1 region of patients with AD in comparison with that in healthy subjects [15]. It has also been reported that AMPA signaling in the central nervous system mediates rapid glutamatergic synaptic transmission, and GluA1-containing synapses are impaired in the early stages of AD [16]. Moreover, an APP/PS1 mouse model was used to show that AMPA activation decreases as Aβ levels in interstitial fluid (ISF) increase [17]. Various animal studies of epilepsy have demonstrated that overexpression of AMPAR and aberrant alterations in AMPAR trafficking result in deficient synaptic plasticity and subsequent neuronal hyperexcitability, which can potentially contribute to epileptogenesis [18,19]. The internalization of AMPARs induced by drugs has been shown to reduce hyperexcitability and spontaneous epilepticus spike frequencies in epilepsy patients [20]. In addition, following administration of the marine toxin domoic acid, a non-desensitizing agonist of AMPARs, patients display temporal lobe epilepsy, which can be explained by the relatively dense expression of AMPARs in the hippocampus [21]. Moreover, postmortem studies of patients with depression have reported lower expression levels of the AMPAR subunits GluA1 and GluA3 in the CA1 region and dentate gyrus in comparison with those in healthy controls. Similarly, several animal studies have found that exposure to chronic stress leads to a decrease in the expression of AMPAR subunits in certain brain regions [22]. A number of preclinical and clinical studies have demonstrated that AMPA has a dose-dependent antidepressant effect, indicating that the development of AMPA potentiators may provide a new approach to treating depression [22,23]. These data suggest that regulating the assembly, expression, and trafficking of AMPARs may prove to be a successful therapy for neurological diseases. This review summarizes the research carried out on AMPAR trafficking-related proteins over the past decade.

## 2. AMPAR Vesicle Formation

Following their synthesis in the GA, AMPARs travel on the cytoskeleton in the form of vesicles and are subsequently inserted into the membrane via fusion. During this process, proteins on both vesicles and the plasma membrane participate in the transportation and localization of AMPAR-containing vesicles. For instance, Rab proteins interact with effector proteins to medicate multiple processes such as vesicle formation, movement, docking, and fusion [24].

### 2.1. Rab11

It has been demonstrated that AMPARs enter cells via grid-mediated endocytosis and are stored in Rab11^+^ endosomal compartments. AMPARs are transported in Rab11^+^ recycling endosomes along microtubule tracks within the dendritic shaft, and removal of these endosomes causes a remarkable decrease in AMPARs and PSD-95 [25]. Following activation during LTP, AMPARs are returned to the synapse via Rab11-mediated endosomal recycling [26].

Rab11, belonging to the Rab GTPase family, is present on the cytoplasmic surface of various intracellular membrane-bound compartments and regulates all stages of intracellular membrane trafficking in eukaryotic cells, including vesicle formation, motility, docking, and fusion [24]. Rab11 is an evolutionarily conserved and widespread subfamily of Rab GTPases consisting of three members: Rab11a, Rab11b, and Rab25 [27].

Rab11 effector proteins discovered to date include the family of Rab11-interacting proteins (FIPs), Rab11BP, myosin-Vb, phosphoinositide 4-kinase β, and Sec15. FIPs act as important effectors of Rab11-driven recycling endosomes, providing functional and regulatory diversity. FIPs can be divided into two categories according to the C-terminal Rab11-binding domain: class I (Rab11FIP1/2/5) has an N-terminal C2 domain, and class II contains an EF-hand domain [28]. N-terminal C2 domains of class I FIPs target Rab11-FIP complexes [24]. FIP2, as a negative regulator of AMPAR membrane trafficking during LTP, is highly colocalized with GluA1. FIP2 contains three distinct domains: an N-terminal phospholipid-binding C2 domain, a C-terminal Rab11-binding domain (RBD), and a central myosin-Vb-binding domain (MBD). Rab11 binding is not required for FIP2 to interact with GluA1, and FIP2–GluA1 dissociation is independent of Rab11 binding, which can prevent AMPAR-containing GluA1 from reaching synaptic membranes without neuronal stimulation [29].

In addition, FIP2 is associated with the tail of myosin-Vb, another Rab11 effector protein. Myosin-Vb forms a homodimer of two identical heavy chains through a coiled-coil region, in which the N-terminus is the head and the tail consists of a series of the coiled proximal tail and distal globular tail domains (GTDs). Myosin-Vb has been shown to act as a dynamic tether to control the direction of Rab11^+^ vesicles containing AMPARs. Rab11a interacts with myosin-Vb and FIP–RBD via the same surface but has a higher affinity for FIP2 [30]. FIP2 combines Rab11a and myosin-Vb through different parts, mediating the binding of myosin-Vb to the membrane. Rab11a binding to subdomain-2 of myosin-Vb–GTD stimulates the ATPase activity of myosin-Vb, which results in the hydrolysis of ATP and the transport of vesicles along actin filaments [31]. The interaction of myosin-Vb with Rab11–FIP2 is involved in maintaining the Rab11a/Rab11–FIP2/myosin-Vb complex and regulating AMPAR trafficking. Myosin-Vb acts as a propulsion motor to propel Rab11^+^ vesicles carrying AMPARs along actin filaments and can resist the movement of organelles along microtubules by anchoring to F-actin filaments. Therefore, myosin-Vb and Rab11–FIP2 are critical for the transport of AMPARs to dendritic spines [32,33]

### 2.2. CaMKII

Studies have shown that GluA2 export from the ER is also controlled by Ca^2+^/calmodulin-dependent regulated kinase II (CaMKII). The release of Ca^2+^ from the ER triggers a signaling cascade that mediates the exit of GluA2 (Figure 1). Following the induction of LTD, the inhibition of Ca^2+^ efflux from the ER in medium-sized spiny neurons stops COPII vesicles containing GluA2 from leaving the ER, thus preventing the trafficking of GluA2 to the plasma membrane [34].

During the process of LTP, glutamate binding stimulates the activation of NMDARs, and the subsequent influx of Ca^2+^ activates CaMKII. CaMKII-mediated phosphorylation of the C-terminal PDZ-binding domain of Stg creates a highly negative charge in its C-terminal tail, which repels membrane lipids and enables the combination of Stg and PSD-95, increasing the localization of AMPARs at the synapse [35]. Following induction via LTP, CaMKII also phosphorylates GluA1, and AMPARs containing phosphorylated GluA1 bind to the Rab11 adapter complex. Rab11 couples to Ca^2+^-activated myosin-Va, which transports these molecules along actin filaments from the dendritic axis to the spine head [36]. Prior to this step, GluA1 from the storage pool binds to FIP2 and separates it from recycling endosomes, which is subsequently dephosphorylated and shed from AMPARs following induction of LTP, allowing AMPARs to bind Rab11 [29].

## 3. AMPAR Vesicle Trafficking

After formation in the ER, AMPAR vesicles need to travel along a “pathway” to a “stop sign” on the synaptic membrane. This “pathway” consists of cytoskeletal proteins that support cells, such as tubulin, actin, and EPB41L1 [37], and the “stop sign” encompasses a group of specific proteins on the membrane that allow AMPARs to anchor in the correct position.

### 3.1. MAP

After formation in the ER, AMPAR vesicles move along cytoskeletal proteins, including actin and tubulin, to the cell membrane. Microtubules, one of the major components of the cytoskeleton involved in the maintenance of synaptic structure and synaptic plasticity, are polymers composed of tubulin proteins [38] that bind to anchored proteins during AMPAR trafficking [39]. During the process of AMPAR trafficking, microtubule-associated protein (MAP) may be involved in the regulation of synaptic plasticity [40]. MAP1A and MAP1B are distantly related proteins that are initially synthesized as polypeptides and subsequently cleaved into a heavy chain (MAP1B-HC/MAP1A-HC) and two light chains (MAP1B-LC/MAP1A-LC). The MAP1B light chain, LC1, can bind both MAP1A and MAP1B heavy chains. The MAP1A light chain, LC2, mainly binds MAP1A-HC; however, it can also bind MAP1B-HC [41]. MAP1B is thought to be involved in vesicle fusion and presynaptic structures [42], and LC1 has been shown to interact with AMPARs via GRIP1. GRIP1 provides a fixed scaffold that connects AMPARs to microtubules and restricts their access to synaptic membranes [39]. MAP1A is expressed in mature neurons and MAP1B in immature neurons. MAP1A has been shown to anchor glutamate receptors to the cytoskeleton by binding PSD-95 and F-actin via the exogenous C-terminal fragment [43]. It has also been demonstrated that MAP1A contributes to presynaptic function by providing an anchoring site for surface Cav2.2, the primary Ca^2+^ entry pathway supporting neurotransmitter release at synapses, to the actin cytoskeleton [44], which facilitates vesicle trafficking of AMPARs. Although MAP1A and MAP1B can bind individually to microtubules and microfilaments, mediating or regulating the interaction between axonal microtubules and actin filaments, the role of their association in AMPAR trafficking remains unclear.

### 3.2. Actin

The abundance of F-actin makes synapses highly dynamic and variable [45]. RIL, an actin cytoskeleton-associated protein, contains a PDZ domain at the N-terminus and an LIM domain (Lin-11, Isl1, MEC-3) at the C-terminus [46]. RIL binds to the C-terminus of GluA1 and the microfilament, cross-linking α-actinin-2 to GluA1 and facilitating the binding of AMPARs to actin. Thus, RIL mediates the interaction between endosomal GluA1 and actin filaments. AMPAR-containing vesicles are actively transported along the actin microfilament to the plasma membrane via myosin-Vb, suggesting that myosin-V plays a crucial role in AMPAR trafficking [47]. Various myosin isoforms are involved in AMPAR trafficking. Myosin-Va and Rab11 are involved in the short-term transportation of GluA1 from the dendritic axis to the spine head [48,49]. Besides, an increase in the transport of the driver protein myosin-Vb enhances the accumulation of AMPARs [46,47]. ARP2/3 (Actin-related protein 2/3, a major factor in the formation of branched actin networks [50]) forms complexes with F-actin and GluA2 via the PDZ domain, BAR domain (Bin/Amphiphysin/Rvs), and C-terminus of PICK1 [51]. ARP2/3 activation is highly regulated in vesicle trafficking and occurs at appropriate times and subcellular locations [51]. In particular, PICK1 is competitively bound by the upstream signaling molecule N-WASP, which inhibits ARP2/3-mediated microfilament nucleation and polymerization [52] and assists in receptor endocytosis and the binding of α-actinin to the N-terminus of PSD-95, ensuring binding of the PSD95–AMPAR complex to the PSD [53].

## 4. AMPAR Anchoring at Postsynaptic Sites

AMPAR-containing vesicles are transported along the actin filaments to the postsynaptic density (PSD), which is a highly organized scaffold protein network composed of membrane receptors, signaling molecules, and cytoskeletal components [54].

PSD is a unique structure located at the postsynaptic membrane of neurons and is composed of postsynaptic density protein 95 (PSD-95; also known as synapse-associated protein 90, SAP-90), postsynaptic density protein 93 (PSD-93), synapse-associated protein 97 (SAP-97), and synapse-associated protein 102 (SAP-102). These proteins belong to the membrane-associated guanylate kinase (MAGUK) protein family, which contains three PSD-95/Dlg/ZO-1 (PDZ) domains at the N-terminus and Src homology 3 (SH3) and inactive guanylate kinase (GK) domains at the C-terminus [55]. Among these three domains, the PDZ domain is the most important protein–protein interaction domain [56], and its general function is to anchor proteins in appropriate cell compartments, thereby promoting protein scaffolding, signaling, and transportation [57]. PSD-95 is one of the most abundant proteins in the PSD. Despite lacking protein activity itself, the interaction of its PDZ domain with AMPARs via TARPs can effectively control the number of AMPARs in the PSD and mediate their localization at the membrane [54,58].

### 4.1. Stargazin

Stargazin (Stg) is a voltage-dependent γ-2 subunit of calcium ion channels and the most well-characterized member of the TARP protein family, which is composed of transmembrane proteins that stabilize the lateral diffusion and synaptic localization of AMPARs [59]. A PDZ ligand at the C-terminus of Stg interacts with PSD-95 and effectively stabilizes the localization of AMPARs at the neuronal membrane [60]. In fact, the cytoplasmic tail of Stg is a key domain for AMPAR trafficking and localization, while its ectodomain is responsible for controlling channel properties and shaping synaptic responses [61]. In addition to participating in AMPAR trafficking, Stg is also involved in the recycling of AMPARs to synaptic surfaces. Following glutamate binding to AMPARs, Stg is shed, which stimulates the internalization of AMPARs [62]. Studies have shown that the absence of AMPARs at the synapse is mainly attributed to a lack of PSD-95 [63,64]. PSD-95 binds directly to Stg to facilitate interaction with the GluA1 subunit of AMPARs, regulating synaptic plasticity and determining the number, size, and shape of dendritic spines for synaptic transmission [65].

### 4.2. SAP-97

Another interacting protein of AMPARs is SAP-97, which participates in the early secretory pathway to promote the maturation of AMPARs [66], unlike PSD-95, which mediates the function of mature synapses [67]. SAP-97 interacts with the PKA-anchored molecule, AKAP-79. Moreover, SAP-97 also binds to GluA1 via the PDZ domain, which results in the recruitment of AKAP-79 to the GluA1 subunit, enhancing Ser^845^ phosphorylation of GluA1 and regulating the processes of LTP and memory retention [68]. Furthermore, overexpression of SAP-97 can rescue the reduction in AMPAR transmission caused by the loss of PSD-95 [69]. However, the association between these proteins may also be involved in AMPAR trafficking, with the interaction being mediated by the binding of the N-terminal segment of SAP-97 to the SH3 domain of PSD-95. Overexpression of PSD-95 triggers the accumulation of SAP-97 in dendritic spines, which can be completely inhibited by overexpression of the SH3 domain of PSD-95. Furthermore, overexpression of PSD-95 induces the recruitment of GluA1-containing AMPARs, which is strongly inhibited by co-expression of the SAP97 NTD and the PSD-95 SH3 domain [70]. In summary, the interaction between the NTD of SAP-97 and the SH3 domain of PSD-95 is sufficient for the synaptic accumulation of GluA1-containing AMPARs but may also interfere with other physiological activities involved in AMPAR trafficking [71].

MAGUK family members interact with AMPARs via the PDZ domain. SAP-97 binds directly to the GluA1 subunit of AMPARs, while PSD-95 requires Stg for the interaction with AMPARs [72]. Moreover, palmitoylation at the N-terminus of PSD-95 is important for AMPAR targeting, whereas SAP-97-mediated targeting of AMPARs is dependent on the alternatively spliced region located between the SH3 and GK domains (I3 domain) [73]. In addition, the I27 domain of SAP-97 at its N-terminus is responsible for rescuing AMPAR-mediated decreased levels of EPSCs induced by PSD-95 knockdown [74]. It appears that all domains of SAP-97 participate in AMPAR trafficking.

It is noteworthy that the recycling and redistribution of AMPARs rely not only on members of the MAGUK family but also on certain small molecules [75], such as postsynaptic adhesion neuroligins. With these binding partners, MAGUKs can effectively organize glutamate receptors and other scaffolding molecules at the PSD [76,77].

### 4.3. PKA

**Figure 2:**
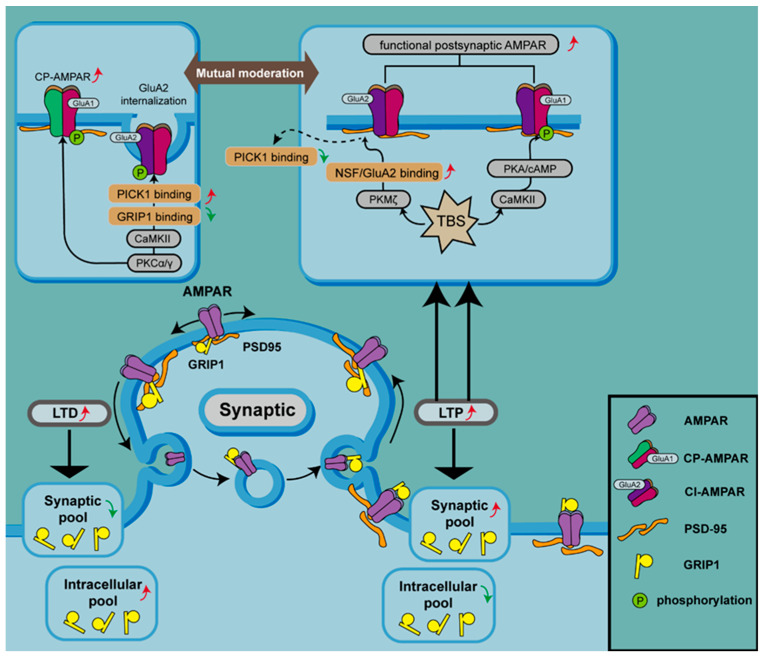
Kinases regulate AMPAR expression via phosphorylation and the intracellular AMPAR recycling pool.

Protein kinase A (PKA) and C (PKC) are downstream effectors of signaling pathways mediated by G protein-coupled receptors. While PKA is mainly involved in signal transduction cascades with cAMP as the secondary messenger, PKC is activated by diacylglycerol (DAG) produced in the phosphatidylinositol signaling pathway. PKA binds to and phosphorylates the Ser^845^ site in GluA1, promoting the phosphorylation of GluA1 at the synaptic membrane and specifically increasing AMPAR expression at the postsynaptic membrane [78]. However, not all LTPs are induced via PKA-mediated phosphorylation of GluA1, nor does phosphorylation by PKA lead to an increase in the expression of all AMPAR types. AMPARs lacking GluA2 are converted to calcium-permeable (CP) AMPARs, while those containing GluA2 are converted to calcium-impermeable (CI) AMPARs. LTP can be divided into early LTP (E-LTP), which is insensitive to PKA and protein synthesis, and late LTP (L-LTP), which is the opposite. An increase in AMPAR insertion into the postsynaptic membrane leads to the induction of E-LTP, which requires the activation of CaMKII. E-LTP can be triggered by one episode of theta burst stimulation (TBS), which can induce the production of cAMP by regulating Ca^2+^-sensitive adenylyl cyclase. L-LTP is enhanced following increased expression of cAMP, a second messenger that subsequently activates PKA. The anchoring of PKA through A-kinase anchoring protein (AKAP) coordinates the incorporation of CP-AMPARs at synapses and regulates the phosphorylation of GluA1 at Ser^845^, which leads to the insertion of CP-AMPARs into the plasma membrane (Figure 2) [79,80,81]. In contrast, disruption of AKAP-PKA anchoring prevents the recruitment and incorporation of CP-AMPARs and boosts the expression of CI-AMPARs, which reduces synaptic AMPAR activity [82]. This suggests that CP-AMPARs are present transiently during LTPs and can be replaced by CI-AMPARs [83], depending on the phosphorylation of PKA. 

The GluA1 subunit of AMPARs can be phosphorylated by CaMKII, PKC, and PKA, which contributes to an increase in the level of AMPAR on the synaptic membrane during LTP. PKCα/γ can phosphorylate GluA2, which disrupts the binding of GRIP1 to GluA2 and increases the interaction of GluA2 with PICK1, resulting in the internalization of AMPARs containing GluA2 and an increase in CP-AMPARs at the synaptic membrane. On the contrary, PKMζ participates in LTP since PKMζ causes dissociation of PICK1 from GluA2-containing AMAPRs by disrupting NSF/GluA2 binding. TBS can induce the generation of cAMP, which activates PKA and initiates the anchoring of PKA to AKAP, coordinating the incorporation of CP-AMPARs at the synapse and regulating the phosphorylation of GluA1 at Ser^845^ (top panel). There are two pools of GRIP1 within cells. GRIP1 mediates AMPAR trafficking between synapses and the cytoplasm, regulating synaptic plasticity. During LTP, the synaptic pool of GRIP1 is increased, and synaptic AMPARs are stabilized and upregulated, while intracellular AMPARs are increased during LTD (bottom panel).

### 4.4. PKC and GRIP 1/2

PKC, another protein kinase involved in the above process, is activated by the induction of LTP and phosphorylates GluA1 at Ser^831^ and Ser^818^ and GluA2 at Ser^880^ [84]. PKC also initiates the autophosphorylation of CaMKII [85]. Studies have demonstrated that phosphorylation at Ser^818^, Ser^831^, and Ser^845^ facilitates the insertion of GluA1 into the synaptic membrane, while TBS enhances this phosphorylation at Ser^818^ and Ser^831^. Of note, among the various candidate kinases and mutant fusion proteins, only PKC phosphorylates Ser^818^. Phosphorylation at Ser^818^ is critical for LTP by modifying binding to other proteins involved in AMPAR trafficking, such as 4.1N, AP2, and PIP3 [86].

Activation of one isoform of PKC, PKCα, acts as a trigger for GluA1 phosphorylation at ser^818^ and subsequent insertion of CP-AMPARs into the synaptic membrane [87,88]. Activation of PKCγ, another isoform of PKC, results in phosphorylation of GluA2 at Ser^880^, which disrupts its interaction with synaptic anchoring protein ABP/GRIP, leading to GluA2 internalization [34] and promotion of CP-AMPAR expression on synaptic membranes (Figure 2) [89].

The dominant kinase that maintains L-LTP is PKMζ, an autonomously active isoform of PKC [90] synthesized following LTP induction. Unlike other PKC isoforms, PKMζ promotes NSF/GluA2-dependent AMPAR trafficking and enhances synaptic transmission through NSF to disassemble PICK1- and GluA2-containing receptors, which leads to an increase in functional postsynaptic AMPARs (Figure 2) [91].

Additionally, it has been found that although AKAPs often bind with PKA to regulate AMPAR trafficking, certain AKAPs, such as AKAP79 [92], AKAP12 [93], and AKAP149 [94], can also regulate PKC. The AKAP79 that functions in PKA phosphorylation of GluA1 was shown to have clearly characterized interactions with the catalytic core of PKC [92,95], binds and inhibits the conserved catalytic core of PKCbetaII, and coordinates the subcellular localization of PKC at the postsynaptic site of neurons. Although the impact of the interaction between PKC and AKAP79 on the function of AMPAR remains to be elucidated, the function of AKAP may involve regulating the balance between PKA and PKC phosphorylation of AMPAR subunits, potentially contributing to a more coordinated regulation of AMPARs at the postsynaptic membrane.

Glutamate receptor-interacting protein 1 (GRIP1) is a scaffold protein containing seven PDZ domains, the fourth and fifth of which interact directly with the C-termini of GluA2 and GluA3, respectively. GRIP1 does not bind to the GluA1 subunit but regulates the expression of GluA1 by controlling the levels of GluA1/2 heterodimers [96]. The binding affinity of GRIP1 for GluA2 is largely influenced by the phosphorylation of GluA2 at Ser^880^ and Tyr^876^. PKC phosphorylates GluA2 at Ser^880^, which disrupts the binding of GRIP1 to GluA2 and, in turn, increases the interaction of GluA2 with PICK1, promoting AMPAR internalization [97] and increasing the surface expression of GluA2 [98].

There are two pools of GRIP1 within cells: the plasma-membrane-associated GRIP1 pool can stabilize surface AMPARs, while the intracellular pool retains AMPARs in intracellular compartments. This mechanism regulates synaptic AMPAR trafficking. During LTD, GRIP1 is removed from the synapse and accumulates in the cytoplasm, resulting in a decrease in AMPAR levels at the cell surface [96].

In homeostatic synaptic upscaling, the synaptic pool of GRIP1 is increased and synaptic AMPARs are stabilized and upregulated, leading to additional trafficking, accumulation of AMPARs at the plasma membrane, and a decrease in intracellular GRIP1–AMPAR interactions (Figure 2) [96,99].

### 4.5. EPH41L1

Erythrocyte membrane protein band 4.1-like 1 (EPH41L1), also called protein 4.1N, is the third discovered cytoskeletal protein 4.1 superfamily member, which links transmembrane proteins to the actin cytoskeleton [100]. There exist three highly conserved domains in the protein 4.1 superfamily: the FERM (4.1-ezrinradixinmoesin) domain, the SAB (spectrin-actin binding) domain, and the CTD (C-terminal domain) [101]. Unlike other 4.1 homologs (4.1B, 4.1G, and 4.1R), the SAB domain of 4.1N does not combine with spectrin or actin. Instead, it interacts with different molecules and other types of proteins through its CTD and FERM domains, which play important roles in modifying synaptic plasticity and synaptic transmission. During AMPAR trafficking, 4.1N is required for GluA1 insertion and directly interacts with the MPR (membrane proximal region) of GluA1 [45]. Meanwhile, 4.1N is associated with the actin cytoskeleton [102]. GluA1 can be anchored to PSD through SAP97 and fixed to the actin cytoskeleton through 4.1N. In this way, the three proteins form a complex, cooperating in the regulation of the synaptic localization and immobilization of AMPARs [103]. Once AMPARs are inserted into the plasma membrane, PKC phosphorylates GluA1 at Ser^816^ and Ser^818^, which enhances the binding of 4.1N to GluA1 and promotes the insertion of GluA1 [104].

## 5. AMPAR Vesicle Fusion

Numerous studies have shown that the LTP process can be induced by the accumulation of AMPARs at synapses. AMPAR-containing vesicles move along actin filaments to non-synaptic sites at the membrane and undergo membrane fusion by exocytosis, which mainly consists of vesicle anchoring and insertion of AMPARs into the membrane. The soluble N-ethyl maleimide-sensitive factor (NSF) attachment protein (SNAP) receptor (SNARE) complex mediates membrane fusion during exocytosis [105].

### 5.1. SNARE Complex

LTP induction requires NMDAR activation, which promotes calcium influx and subsequent SNARE-dependent membrane fusion of AMPAR-containing endosomes. The SNARE complex is typically composed of SNARE proteins localized in vesicles (v-SNARE) and target membranes (t-SNARE), which are essential for the membrane fusion process [106]. As AMPAR-containing vesicles move along actin filaments to the locality of the plasma membrane, the v-SNARE protein synaptobrevin-2 (Syb-2) on the vesicle interacts with two t-SNARE proteins, syntaxin-3 (Stx-3) and SNAP-47, on the plasma membrane to form a SNARE complex; thus, vesicles containing AMPARs are anchored to the synaptic exocytosis site [105]. Initially, Stx-3 binds to the SM (Sec1/Munc18-like) protein Munc-18, which organizes the assembly of v-SNAREs with t-SNAREs [107]. After assembly, SNARE complexes bind to Complexin, producing an activated but frozen state [107]. Upon NMDAR activation and subsequent calcium influx, the calcium sensor Synaptotagmin-2 (syt2) on AMPAR-containing vesicles receives Ca^2+^ signals and removes Complexin, promoting membrane fusion [108] and the insertion of AMPARs into the synaptic membrane. Interestingly, a recent study showed that SNARE, as an essential mediator of vesicle transport, is not only involved in membrane fusion but also in the sorting and targeting of various proteins, which requires the N-terminal domain of the respective SNARE proteins [109].

NSF is a hexamer ATPase that participates in the recycling of SNARE complexes. NSF promotes the disassembly of the SNARE complex and accelerates its recycling by hydrolyzing ATP to increase the abundance of AMPARs at the membrane. In addition, loss of NSF results in decreased expression of GluA1–3 at the membrane. Since NSF binds directly to the C-terminal domain of GluA2 and does not interact with GluA1 or GluA3, decreases in the expression of GluA1–3 may manifest as a reduction in GluA2 at the membrane [110]. Multiple studies have shown that blocking the interaction between NSF and GluA2 can reduce the amount of GluA2 in synapses [111,112]. Since NSF can disassemble the GluA2–PICK complex in vitro, it is speculated that NSF promotes the membrane expression of AMPARs by disassembling protein complexes that impede their membrane insertion [113]. Furthermore, post-translational modifications also influence the function of NSF, which in turn affects the presence of AMPARs at the synaptic membrane. Nitrosylation of Thorase at the C^137^ residue can transnitrosylate NSF, which improves the affinity of NSF for GluA2 and stabilizes the Thorase–NSF–GluA2–GRIP1 complex while promoting disassembly of the GluA2–PICK1 complex, inhibiting endocytosis of GluA2, and increasing the expression of GluA2 at the synaptic membrane [114].

### 5.2. AP-2

During LTD, AMPARs are removed from the cell surface by endocytosis, after which a pool of AMPARs in the endosomal recycling compartment can be transported to the dendritic spine and spread laterally to the PSD [115].

The adaptor protein 2 complex (AP-2 complex) is mainly involved in the induction of AMPAR internalization; the μ2 subunit of AP-2 binds directly to a basic motif with high affinity, which is present in the cytoplasmic tail (CT) of GluA1–3 and in the presynaptic vesicle protein Synaptotagmin 1 [116]. It is noteworthy that the binding site for AP-2 in GluA2 overlaps with that for NSF [117], indicating that AP-2 may compete with NSF for the binding of GluA2. The binding of NSF to GluA2 enhances AMPAR expression at the synaptic surface by facilitating the delivery of GluA2 to the postsynaptic membrane [111], while AP-2 promotes the endocytosis of AMPARs. In addition, studies have demonstrated that AP-2 interacts directly with activity-regulated cytoskeleton-associated protein (Arc), also known as the activity-regulated gene of 3.1 kb (Arg3.1), which facilitates AP-2 interaction with GluA2 at the plasma membrane. AP-2 recruits clathrin to the membrane, and Arc subsequently binds endophilin and dynamin to promote endocytosis of vesicles containing AMPARs [118]. 

Based on the above summary of the proteins related to AMPAR trafficking, including their functions and regulatory mechanisms described in the latest studies, a comparatively complete AMPAR trafficking mechanism could be described (Table 1).

AMPARs, synthesized in the ER, are glutamate ionotropic receptors located in the PSD. In the absence of neuronal stimulation, FIP2 binds to the GluA1 subunit of AMPARs to prevent entry into the synapse [29]. Following LTP induction, glutamate binds to and activates NMDARs [119], which results in Ca^2+^ entering postsynaptic cells and activating CaMKII to phosphorylate GluA1 and Stg (γ_2_). Vesicles containing phosphorylated GluA1 subunits bind to the Rab11 adapter complex, and Ca^2+^-sensitive Myosin-Va carries this complex and AMPAR-containing vesicles along the actin filaments to the dendritic spine head. Ca^2+^-activated Myosin-Vb transports AMPARs to the exocytosis site along actin filaments [120,121]. PKC phosphorylates the Ser^816^ and Ser^818^ sites of the GluA1 subunit and promotes the binding of GluA1 to EPH41L1, allowing AMPARs to anchor to the actin cytoskeleton [78]. CaMKII-mediated phosphorylation of the C-terminal PDZ-binding domain in Stg produces a highly negatively charged C-terminal tail, which is subsequently unfolded and conducive to its binding to the PDZ region of PSD-95, promoting the localization of AMPARs at synaptic exocytosis sites [122]. The surface of vesicles containing AMPARs usually contains Syb-2. As the vesicles are transported to the exocytosis site, Syb-2, Stx-3, and SNAP-47 are bound together [105] to facilitate the anchoring of the vesicles containing AMPARs to the synaptic membrane. Syt-2 on vesicles binds Ca^2+^, which promotes membrane fusion mediated by the SNARE complex, thereby inserting AMPARs into the synaptic membrane [123].

## 6. The Actin Cytoskeleton That Facilitates AMPAR Trafficking

AMPAR trafficking involves multiple dynamic processes of vesicle cycling, all of which involve the actin cytoskeleton, which is highly dynamic and regulated. Actin dynamics generate forces that manipulate the membrane during vesicle biogenesis and are also used to propel vesicles through the cytoplasm to their destinations. Actin and actin-binding proteins (ABPs) are highly enriched at the postsynaptic density (PSD), where actin cytoskeleton dynamics maintain dendritic spine morphology during neuronal development and undergo expansion and contraction during synaptic activity changes [124]. Interestingly, as summarized, the actin cytoskeleton plays a critical role in controlling the dynamic localization of AMPAR by regulating multiple points in the transport pathway, such as the binding between Rab11-FIP2 and myosin-Vb during AMPAR vesicle formation mentioned earlier, the binding of MAP1A to PSD-95 and F-actin during vesicular transport, and the membrane insertion of AMPAR mediated by EPH41L1, a member of the actin cytoskeleton protein family, during AMPAR post-synaptic anchoring.

Furthermore, some other proteins involved in AMPAR transport have also been found to have an inseparable relationship with the actin cytoskeleton. For example, the actin-regulatory protein cortactin acts as an intracellular sorting mediator for AMPARs. Disruption of the binding between GluA2 and cortactin in neurons results in the targeting of GluA2/A3 receptors to lysosomes and subsequent degradation, leading to the loss of surface and synaptic GluA2 under basal conditions and blocking subsequent LTD expression [125]. Furthermore, studies have shown that the absence of cofilin, an actin-depolymerizing factor, leads to increased stability of the actin cytoskeleton, facilitating the formation of larger spines and preventing the recruitment of AMPARs to synapses following synaptic activity [126]. Although the exact molecular relationship between actin dynamics and synaptic AMPAR insertion remains unclear, it is evident that actin stability, AMPAR postsynaptic expression, and trafficking are interconnected.

The first step in reshaping the spine actin network is to dissociate actin filaments that are typically crosslinked by unphosphorylated CamKIIβ [127]. CamKIIβ stabilizes the actin network by preventing the binding of other ABPs by holding two filaments (or filaments and proteins at PSD) together. During LTP induction, CaMKIIβ is activated by calcium/calmodulin binding and CaMKIIα phosphorylation. Then, CaMKIIβ dissociates from g-actin, making g-actin monomers available for cytoskeleton network growth. In addition, the dissociation of CaMKIIβ and α-actinin4 (another crosslinking protein that binds to actin and PSD proteins) allows other ABPs to bind actin [128]. These results suggest that CamKII may play more complex roles in the regulation of spine morphology and synaptic plasticity. AMPAR assembly occurs in the ER and is transported to the cell surface by vesicles. Studies have shown that the increase in synaptic strength is correlated with stable anchoring of the ER [129]. In the mammalian central synapse, only a small fraction of spines contain ER at any given time point (15–50% [130,131]), but this dendritic ER is highly dynamic and explores most spines transiently over time [129]. MyoVa plays the procedural organelle transporter role in dynamically delivering ER into spines, during which caldendrin converts myosin into fixed F-actin scaffolds that allow for the positioning of ER tubules in dendritic spines and the formation of spine apparatus [132]. This implies that actin cytoskeleton dynamics-mediated dendritic ER regulation is also an important regulatory factor for AMPAR transport.

It is evident that the role of the actin cytoskeleton in AMPAR transport may be far more complex than the listed ones, where many more intricate relationships may exist. For many years, research on synaptic functional plasticity and morphological plasticity has supported the hypothesis that the atypical environment of dendritic spines is a small, closed compartment with very high concentrations of dynamic actin filaments. As LTP involves inserting new AMPARs into the “slots” composed of various structural and anchoring proteins, such as PSD-95 situated at PSD, it is necessary to change spine size to provide space for new AMPARs [133], requiring the reshaping of the actin filament branching network to rebuild the spine actin cytoskeleton [134]. This indicates that the induction protocol for synaptic plasticity indeed induces rapid reshaping of the spine actin cytoskeleton [135] to enhance postsynaptic responses. However, mechanism details remain far from clear. In fact, due to extensive crosstalk between different actin regulation molecules/pathways and bidirectional interactions and activations between memory molecules and actin, delineating the specific functions of individual actin-binding molecules involved in synaptic formation and AMPAR transport still poses a formidable challenge. Although some studies have proven that actin-binding proteins or actin-regulatory proteins associated with AMPAR subunits or postsynaptic scaffolding are critical regulatory factors for AMPAR transport, it is necessary to gain a more comprehensive understanding of the spatio-temporal regulation of actin dynamics related to AMPAR and PSD.

## 7. AMPAR Trafficking in Neurological Diseases

Due to the crucial role of AMPAR in the nervous system, numerous studies have demonstrated that targeting AMPAR can influence the development of related neurological diseases by utilizing various AMPAR activators or antagonists. However, most of these studies have focused only on directly supplementing or antagonizing AMPAR and observing disease progression, without further investigating the specific processes and mechanisms of AMPAR transport involved in various diseases. This simplistic regulation of AMPAR may not achieve long-lasting effects. However, as research on AMPAR in diseases continues to grow, there is gradually more explanation of the transport mechanisms of AMPAR in diseases, which helps us to understand the progression of diseases in more detail and develop more effective treatment or prevention methods.

AMPAR-related vesicle formation and transport processes are highly susceptible to disease. Rab11 functions as an important protein for endocytosis and intracellular vesicle recycling and plays a crucial role in various neurological diseases, particularly those associated with protein pathological aggregation. In Huntington’s disease (HD), huntingtin protein (HTT) regulates Rab11-dependent transport, which is involved in maintaining dendritic spine size [136]. Mutant HTT fragments disrupt Rab11-mediated endocytic transport and impair the membrane localization of GluA1, while overexpression of *Rab11* restores HD-related phenotypes in a Drosophila model [137]. Early Alzheimer’s disease (AD) exhibits dysfunctions in the endocytic cycle, and its impairment of the intracellular recycling system is closely linked to Rab11 [138]. Studies have demonstrated that BIN1, a risk factor for late-onset Alzheimer’s disease (LOAD), co-localizes with Rab11, forms complexes with the AMPAR subunit GluA1, and contributes to maintaining normal spine morphology and AMPAR-mediated synaptic transmission in Alzheimer’s disease [139]. Interestingly, only AMPAR containing the GluA1 subunit colocalized with Rab11-positive endosomes during cerebral ischemia and reperfusion (OGD/R), while the GluA2 subunit was not present in Rab11-positive endosomes. This may indicate the differential membrane surface recycling mechanisms for GluA1 and GluA2 during the OGD/R process [140]. In addition, Rab11 also plays an important role in Parkinson’s disease (PD) induced by α-synuclein aggregation. In a Drosophila model of PD, Rab11 rescues the increase in synaptic vesicle size induced by α-synuclein and collaborates with LRRK2 to regulate synaptic vesicle cycling, possibly related to the transport cycle of AMPARs [137,141]. CaMKII, as a direct downstream protein of neuroexcitotoxicity caused by Ca^2+^ influx, has also been studied in neurological disorders involving AMPAR. And one study clarified the mechanism by which oligomeric A β regulates CaMKII and then regulates synaptic AMPAR stability in AD. Oligomeric Aβ triggers non-autonomous activation of CaMKII in a competitive manner, preventing the auto-phosphorylation activation of CaMKII at the T286 site induced by LTP, leading to dendritic spine loss through destabilization of surface AMPARs [142]. Intellectual disability is closely associated with synaptic plasticity mediated via AMPAR. Research has identified 19 variants of *CAMK2A* or *CAMK2B* in the exome sequencing of 24 individuals with intellectual disabilities. The mutated CAMK2 shows different levels of abnormal autophosphorylation, which may lead to learning and synaptic plasticity impairments in the downstream AMPAR-dependent signaling pathway, potentially being a key factor in the learning and memory deficits mediated by developmental delay/ID [143].

Surprisingly, TARPs, such as stargazin, which stabilizes AMPARs, have been found to directly participate in disease processes in various diseases. Brain quantitative proteomic analysis of AD models showed that stargazin gradually disappears from AMPAR complex sets with increasing Aβ levels, accompanied by impaired glutamatergic synaptic plasticity. Supplementing with stargazin can restore impaired excitatory synaptic transmission in AD [144]. In patients with intellectual disabilities, a stargazin V143L missense mutation disrupts the interaction between stargazin and AMPAR, resulting in AMPAR transport defects, synaptic dysfunction, and the manifestation of cognitive and social behavioral impairments associated with intellectual disability [145]. *HTT* mutations in HD have been found to weaken the interaction between HTT and stargazin-PSD95, accompanied by a surface diffusion imbalance of AMPARs [146]. Stargazin exhibits higher S-nitrosylation in the ventromedial prefrontal cortex (vmPFC) of anxious mice. Injection of an exogenous stargazin (C302S) mutant can alleviate S-nitrosylation, rescue GluA1 and AMPAR-mediated synaptic transmission, and improve anxiety-like behaviors [147]. Furthermore, dysregulation of stargazin expression has been found in the brains of patients with schizophrenia and affective disorders [148]. Selective deletion of a portion of TARP γ-8, which interacts exclusively with AMPAR within the TARP family, partially restores neuronal loss in epileptic seizures [149]. These findings suggest that the collaborative regulation of synaptic function by stargazin and AMPAR may be involved in the pathogenesis of various neurological disorders.

OGD/R-induced impairment of AMPAR trafficking was shown to be PICK1-dependent [150]. Disrupting the interaction between PICK1-GluA2 and the c-terminal GluA2 peptide (EVKI) can effectively attenuate OGD-induced GluA2 internalization [150]. Additionally, other research has suggested that AP is also involved in this process, as AP interacts with PICK1 to mediate the OGD-induced endocytosis and degradation of GluA2 [151]. The interaction between PICK1 and GluA2 also plays a critical role in neuropathic pain, affecting hyperalgesia in sciatic nerve constriction injuries [152]. Mutations in *RAB39B*, associated with intellectual disability and epilepsy, can also bind to PICK1 to regulate the AMPAR subtype ratio and alter synaptic activity [153]. Amyotrophic lateral sclerosis (ALS), one of the most common motor neuron diseases, is associated with the loss of function of the *ALS2* gene. Studies have shown that ALS2 interacts with GRIP1 both in vitro and in vivo and colocalizes with GRIP1 in neurons [154], which may be part of the mechanism underlying synaptic transmission impairment in ALS.

Studies on AD have shown that Aβ interacts with PSD-95, whether in the postmortem brains of AD patients or in primary neurons exposed to Aβ oligomers [155]. Therefore, the pathological mechanism of AD may involve Aβ directly interacting with PSD-95 to alter the synaptic localization of AMPAR, leading to synaptic dysfunction. Treatment with the antidepressant thianepine in a mouse model of HD can rescue AMPAR surface imbalance through a BDNF signaling-mediated pathway involving TARP and PSD95, restoring impaired LTP and hippocampus-dependent memory in different HD mouse models [146]. Recently, exome sequencing studies have identified *PSD-95* as a high-risk gene associated with autism spectrum disorders [156]. Behavioral studies have shown that *PSD-95* knockout mice exhibit phenotypes similar to autism [157], suggesting a potential link between the disruption of the PSD-95-AMPAR connection and the pathological mechanism of autism.

HTT controls the transport of AMPARs containing GluA2 along microtubules in dendrites through the motor protein kinesin 5 (KIF5). In HD models, the GluA2/KIF5/HAP1 complex is disrupted, leading to loss of microtubule binding ability and impaired movement of vesicles containing AMPARs along microtubules [158]. Additionally, HTT affects actin dynamics through the LIMK-cofilin pathway, playing a role in recruiting AMPAR to active synapses. Loss of *HTT* results in a disconnection between spine structure and synaptic function, potentially contributing to the development of HD symptoms [126]. Enrichment analysis of hippocampal LTP protein components related to AD has highlighted the critical role of the actin cytoskeleton pathway in hippocampal LTP [144].

## 8. AMPARs PTM

**Figure 3:**
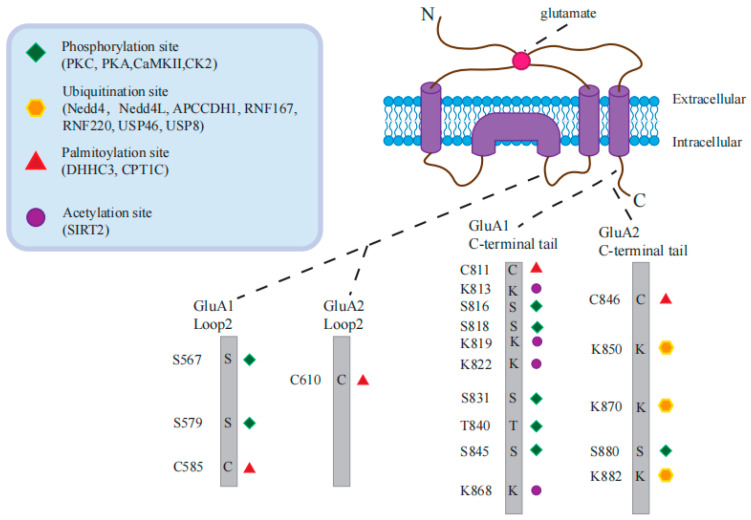
AMPAR post-translational modification sites and related proteins.

In addition to the subunit-specific protein interactions, AMPARs also undergo many post-translational modifications (PTM) that contribute to the regulation of synaptic activity and plasticity (Figure 3). Among the three current major PTMs of AMPARs, phosphorylation and palmitoylation mainly influence receptor channel conductance and intracellular trafficking, while ubiquitination serves as the signal for receptor internalization and proteasomal degradation [4]. Lysine acetylation is a newly described PTM of AMPARs [159], which represents the only known modification of receptors on stable cell membranes and thus has great research potential. In the following sections, we introduce PTM and protein-modifying enzymes involved in the process of AMPAR trafficking.

The AMPAR are substrates for several kinases targeting serine (S) and threonine (T). Also, AMPARs are modified by palmitoylation on cysteines (C) and ubiquitination (UB) on lysines (K). The amino acids (aa) of GluA1-2 loop2 and C-terminal tails targeted by specific PTMs are depicted.

### 8.1. Phosphorylation in AMPAR Trafficking

AMPARs serve as a substrate for various proteins, facilitating the occurrence of synaptic plasticity mechanisms like long-term potentiation (LTP), long-term depression (LTD), and other forms. These proteins, including protein kinase C (PKC), cyclic AMP-dependent protein kinase/protein kinase A (PKA), and calcium/calmodulin-dependent protein kinase II (CaMKII), have been extensively studied by researchers. They interact with the C-terminal region of different AMPAR subunits through various modifications such as phosphorylation and dephosphorylation, thereby regulating the synaptic transport and plasticity of AMPARs.

In particular, phosphorylation of GluA1 at Ser^845^ by PKA and at Ser^831^ by CaMKII or PKC has been extensively studied. PKA-mediated phosphorylation of GluA1 at Ser^845^ has been shown to promote its cell-surface insertion and synaptic retention, whereas dephosphorylation at Ser^845^ is related to receptor endocytosis, LTD, and homeostatic scaling-down [160]. Following the binding of Ca^2+^ to CaMKII, auto-phosphorylation occurs to activate CaMKII, which phosphorylates GluA1 at Ser^831^, not only promoting the insertion of AMPARs into the postsynaptic membrane but also enhancing channel conductivity and eventually facilitating LTP [161]. Moreover, research shows that GluA1 is phosphorylated by CaMKII at Ser^567^, which inhibits the synaptic insertion of GluA1-containing AMPARs under basal conditions [161].

PKC-mediated phosphorylation of GluA1 at Ser^816^ and Ser^818^ enhances its interaction with 4.1N, which promotes GluA1 insertion into the postsynaptic membrane, consequently strengthening LTP. However, palmitoylation of GluA1 at Cys^811^ disrupts its interaction with 4.1N and impairs LTP [102]. PKC-mediated phosphorylation of GluA2 at Ser^880^ interferes with its interaction with GRIP [162]. Moreover, casein kinase 2 (CK2) phosphorylates the loop 1 region of both GluA1 and GluA2 [163], and the mutation of GluA1 at Ser579, the major CK2 phosphorylation site, impairs both the surface and synaptic expression of AMPAR.

Over the years, a plethora of data has continually indicated an important part of AMPAR phosphorylation in the regulation of synaptic expression and dynamic AMPAR changes during synaptic plasticity. Research shows that 4.3% of all GluA1 found in the hippocampus is phosphorylated at Thr^840^; however, GluA1 phosphorylated at Ser^831^ or Ser^845^ represents only 0.18% and 0.018% of the total GluA1, respectively [164]. However, other studies have estimated that closer to 15% of surface GluA1 is phosphorylated at Ser^845^ under steady-state conditions [165]. A possible explanation for this discrepancy is that the phosphorylation state of AMPAR is transient in nature, which suggests that both the spatial and temporal resolution should be considered in order to gain a more accurate measurement.

### 8.2. Ubiquitination in AMPAR Trafficking

Nedd4, Nedd4L, APCCdh1, and RNF167 are the four AMPA receptor-specific E3 ubiquitin ligases that have been shown to control synaptic activity in primary neurons. [166,167,168,169,170]. It has been reported that only two specific E3 ubiquitin ligases, GluA1 and GluA2, which can ubiquitinate in the C-terminal domain, have been identified [171]. Nedd4, Nedd4L, and APCCdh1 regulate GluA1 ubiquitination [166,167,168,169], while RNF167 regulates GluA2 ubiquitination [170]. The GluA1 subunit is primarily ubiquitinated at the C-terminal, while GluA2 is predominantly ubiquitinated at Lys^870^ and Lys^882^ in the same region [172]. In addition, although RNF167 regulates GluA1 and GluA2 expression on the surface of neurons [170], only its ubiquitination function has been studied, and it is unclear whether these E3 ligases have specific selectivity for these two AMPAR subunits. According to statistics, the researchers have found that 83 of the 600 to 700 E3 ubiquitin ligase genes in the genome are associated with brain disease [173]. Nevertheless, with the exception of those extensively studied, the regulation of AMPAR by other E3 ubiquitin ligases remains elusive. A new study [174] found that RNF220, a ubiquitin ligase responsible for AMPAR, induces synaptic deterioration on neuronal surfaces. After depleting RNF220 from neurons in the forebrain, the levels of GluA1 and GluA2 are elevated, leading to an enhancement in AMPAR-mediated synaptic activity. Moreover, neuropathological mutations in RNF220 lead to dysfunctional AMPAR ubiquitination and loss of excitatory synaptic transmission.

It is likely that the process of AMPAR ubiquitination plays a role in both the endocytosis and the sorting of these receptors toward late endosomes. Multiple research teams have gathered evidence supporting the involvement of ubiquitination in controlling the intracellular trafficking of AMPARs to late endosomes [175]. However, there is ongoing debate regarding the cellular significance of GluA1 and GluA2 ubiquitination in mediating AMPAR endocytosis [171,175].

Some evidence exists to support the view that the ubiquitination of GluA1 is an endocytosis signal for AMPARs. For example, the overexpression of *Nedd4-1* in neurons enhances the ubiquitination of GluA1, which results in a decrease in the number of AMPARs at the plasma membrane [166,167,176]. Moreover, the knockdown of *Nedd4-1* increases the rate of GluA1 internalization [176]. Similarly, overexpression of *USP46* downregulates the ubiquitination of GluA1 and reduces the accumulation of internalized GluA1 in neurons [177]; therefore, it has been suggested that ubiquitination of AMPARs is necessary for internalization and thus occurs prior. Another view suggests that endocytosis precedes the binding of ubiquitin to AMPAR. For example, inhibition of enzymes critical to membrane fission during endocytosis or of the formation of clathrin-coated pits has the potential to impede the ubiquitination of all subunits of AMPARs in cultured neurons upon exposure to either AMPA or bicuculine [178]. In addition, expression of GluA1-4KR ubiquitin-deficient mutants does not affect the surface localization of AMPARs or their agonist-induced internalization [175]; however, these AMPAR vesicles were wrongly classified as recovered endosomes and returned to the plasma membrane, indicating that ubiquitination is not necessary for the internalization of AMPARs. The role of AMPAR ubiquitination in its endocytosis is still questionable; nevertheless, the consensus is that AMPAR ubiquitination controls its intracellular sorting into late endosomes for subsequent degradation.

In addition, there also exist deubiquitinating enzymes (DUBs) for AMPARs, which facilitate the elimination of covalently bound ubiquitin, thus overseeing ubiquitin signaling and upholding a liberated ubiquitin reservoir within cells. Ubiquitin-specific protease 8 (USP8) is a crucial controller of the endosomal sorting complex required for the transport (ESCRT) pathway. In cultured neurons, an elevated presence of USP8 decreases the level of agonist-induced AMPAR ubiquitination, resulting in heightened expression of surface GluA1 and synaptic AMPARs [179]. The effectiveness of this impact is nullified when the catalytic cysteine residue in the USP domain undergoes mutation, underscoring the critical need for USP8 deubiquitinating activity in regulating AMPAR trafficking. USP46 is enriched at synapses and expressed ubiquitously in the brain. In a similar manner to USP8, the reduction in USP46 through knockdown enhances basal GluA1 ubiquitination while diminishing its neuronal expression [177]. In addition, GluA1 internalization decreased with USP46 overexpression, and AMPAR surface expression increased, consistent with its DUB function.

Moreover, since AMPARs are widely expressed in the brain and the subunit composition of the tetrameric complex is different across brain regions [180,181], it becomes intriguing to investigate if these ubiquitin ligases exclusively operate within the brain, serving as a pivotal mechanism to tightly regulate AMPAR ubiquitination and function.

### 8.3. Palmitoylation in AMPAR Trafficking

Another form of AMPAR modification of AMPARs is the covalent attachment of the lipid palmitate via thioester bonds at their intracellular cysteine residues. Protein S-palmitoylation, a post-translational modification, resembles phosphorylation in its reversible and labile nature [182]. The catalysis of this process is facilitated by palmitoyl acyltransferases (PATs) containing conserved Aspartate–Histidine–Histidine–Cysteine (DHHC) motifs, while the reversal is carried out by depalmitoylating enzymes like acyl protein thioesterases and palmitoyl protein thioesterases.

Palmitoylation of GluA takes place in two separate groupings: one found within the second transmembrane domain (TMD2) and another at the C terminus in the juxtamembrane domain near the fourth TMD (TMD4). The palmitoylation process of GluA1 at TMD2 is facilitated by DHHC3, a Golgi-resident PAT. Elevated levels of palmitic acid and DHHC3 cause GluA1 palmitoylation, promoting GluA retention in the Golgi and suppressing its activity-dependent delivery to the plasma membrane [183], implying that depalmitoylation of AMPARs at the Golgi apparatus would be a signal for their release to the cell surface.

Specifically, depalmitoylation at Cys^585^ in GluA1 favors trafficking to the plasma membrane. Carnitine palmitoyltransferase 1C (CPT1C) belongs to the family of carnitine long-chain acyltransferases and is located in the ER of neurons [184]. It has been demonstrated that CPT1C-mediated enhancement of the cell surface expression of GluA1-containing AMPARs is dependent on the palmitoylable Cys^585^ residue [185], indicating that alterations in the palmitoylation condition of GluA1, which is facilitated by CPT1C, could potentially account for this observed outcome.

In the ER, AMPARs are positioned with their palmitoylation sites facing the cytoplasmic side [186], and the catalytic domain of CPT1C also faces the cytoplasm; therefore, this topology allows contact between the two protein domains. Thiolipase activity is performed by a catalytic triad of the CPT1C domain that transports the palmitoyl moiety from the substrate (GluA1) to the carnitine molecule, leading to the depalmitoylation of GluA1, which increases the surface expression of AMPARs [187]. Since CPT1C fails to promote GluA2 homomeric AMPAR trafficking [185], the identity of the depalmitoylating enzyme for GluA2 in the ER remains to be elucidated.

In addition, GluA1 palmitoylation at Cys^811^ has been shown to modulate synaptic plasticity in mice. In a recent study, researchers investigated the impact of GluA1 palmitoylation on AMPAR function in the hippocampus of live animals [188]. Specifically, GluA1 palmitoylation-deficient knock-in mice, in which the C-terminal palmitoylation site of GluA1 (a cysteine at residue 811) was replaced by serine (C811S), had a similar AMPAR function to normal mice, while abnormal GluA1 palmitoylation could lead to brain overexcitation. In addition, the researchers found that normal and GluA1 mutant mice had similar APMAR functions, suggesting that C-terminal palmitoylation is optional for baseline AMPAR transport, further revealing that GluA1 palmitoylation may be primarily involved in threshold regulation of normal brain overexcitability.

### 8.4. Acetylation in AMPAR Trafficking

Acetylation, a post-translational modification of proteins that was not widely studied until recent years, is the covalent attachment of acyl-CoA compounds to specific residues, typically lysine, under the action of acetyltransferase. Acetylation occurs on histone and non-histone proteins; however, the majority of current research focuses on the acetylation of histones.

A recent study established that lysine acetylation is a novel post-translational modification of AMPARs and has been shown to be able to prolong the AMPAR half-life and stabilize the receptor modification on cell membranes, thus playing a crucial role in the brain. Acetylation of AMPAR appears to repel ubiquitination. When AMPAR is acetylated, ubiquitination does not occur, possibly because the AMPAR subunit is protected when the acetyl group re-places the lysine site [167]. Interestingly, this competitive relationship with AMPAR was also observed in neuron culture. When AMPAR acetylation levels increased, ubiquitination levels decreased, and vice versa. However, differences in forms of modification are not necessarily mutually exclusive [175], and an AMPAR-specific modification site may favor one modification over another. Briefly, the K868 site, the last lysine, is a preferential site for ubiquitination modifications in comparison with the other sites [167]; therefore, the preferred site for acetylation is probably at the site of another lysine residue or residues. In this case, both forms of modification (acetylation and ubiquitination) could exist on AMPARs together, and the relative weights of acetylation and ubiquitination will directly affect the synaptic transport function of AMPARs. This competition may not only occur between acetylation and ubiquitination but also between other forms of post-translational modification containing phosphorylation.

One study [159] found that AMPARs have a high level of acetylation under basic conditions, which can be effectively regulated by neuronal activity. Increased acetylation results in increased expression of AMPAR at the membrane and enhanced basic synaptic transmission strength both in vitro and in vivo. SIRT2 has been identified as a deacetylase for AMPARs [159]. The homeostatic equilibrium of AMPAR is due to the competition of SIRT2 for lysine residues at the C-terminal of GluA1, resulting in the relative weights of acetylation and ubiquitination of AMPAR reaching balance. Deacetylation of AMPARs via brain-enriched SIRT2 facilitates receptor ubiquitination and leads to receptor internalization and proteasomal degradation; therefore, an in-depth study of AMPAR acetylation-related enzymes is crucial for understanding the regulation of AMPAR turnover, synaptic plasticity, and cognitive brain function.

These studies suggest that cognitive improvement may not be simply dependent on AMPAR accumulation but needs to be assisted by optimizing the kinetics of newly synthesized AMPAR. Therefore, aiming for normalized AMPAR transport capacity and receptor synthesis kinetics rather than simply increasing the total number of AMPARs should be the most effective measure to ameliorate cognitive dysfunction in the brain.

### 8.5. Crosstalk between AMPAR PTMs

The synaptic transport and delivery functions of AMPAR are subject to crosstalk by the functional interactions of different post-translational modifications to precisely regulate AMPAR-mediated synaptic signaling [4]. This conclusion is mirrored by a recent study in which inhibition of GluA1 ubiquitination resulted in elevated levels of PKA phosphorylation at Ser^845^, whereas PKC/CaMKII phosphorylation at Ser^831^ was unchanged [189]. Interestingly, the phosphomimetic S845D mutant negatively regulates AMPAR ubiquitination by disrupting the binding of GluA1 to the ubiquitinating enzyme. These two different modifications of GluA1 regulate the membrane sorting of AMPAR, and therefore, the ubiquitination and phosphorylation reach a homeostatic state that determines the number of AMPARs expressed on the plasma membrane surface. However, it remains to be investigated whether the ubiquitination of AMPAR is interconnected with other post-translational modifications.

In addition to the crosstalk between phosphorylation and ubiquitination of AMPAR, the researchers also identified functional interactions between phosphorylation and palmitoylation. phosphorylation levels near PKC phosphorylation at Ser^816^ and Ser^818^ are positively regulated by depalmitoylation of the C-tail Cys^811^ of GluA1, which in turn cross-talks the 4.1 N with GluA1 to promote the insertion of GluA1 into the plasma membrane, contributing to the regulation of synaptic plasticity [102]. Moreover, AMPAR ubiquitination was found to exist in a communication network with palmitoylation [190] or phosphorylation [191] that regulates signal transduction, thereby finely regulating the synaptic transport of AMPAR. Therefore, it will be crucial to determine whether AMPAR transport and synaptic function are regulated via multiple PTM crosstalks.

## 9. AMPAR PTMs in Neurological Diseases

As scientific research and technology advance, AMPAR PTM has been increasingly studied in neurological diseases. The regulatory potential of AMPAR PTMs on synaptic homeostasis continues to be unveiled. We have summarized the AMPAR PTM that has been clearly implicated in diseases so far (Table 2), providing a better understanding of the study of AMPAR PTMs in diseases.

Phosphorylation, as one of the most extensively studied protein modifications, has been widely discussed in the nervous system. Research on AD indicates that Aβ drives the phosphorylation of the S880 residue in GluA2 during LTD, and the expression of the GluA2 S880E mutant prevents the internalization driven by LTD while also blocking Aβ-induced morphological and synaptic suppression. Simulating this AMPAR phosphorylation produces spine morphology and synaptic suppression similar to Aβ-induced effects [192]. Increased GluR1 membrane insertion and increased GluR2 internalization were detected in the mouse continuous inflammatory pain model, accompanied by increased PKCα-mediated phosphorylation of GluA2 at Ser^880^, and PICK1 in the spinal cord also promoted this process [152]. The use of tPD5, a short peptide inhibitor of PICK1, was found to completely alleviate neuropathic pain in a mouse nerve injury (SNI) model, while also reducing the phosphorylation of GluA2 S880 in the spinal cord, reversing the surface expression of GluA1 and GluA2, and the membrane insertion of CP-AMPARs in neuropathic pain [193]. Additional study suggested that persistent phosphorylation of AMPARs mediated by PKCγ in postsynaptic density (PSD) of spinal dorsal horn neurons may contribute to neuropathic pain due to the loss of dephosphorylation activity of calcium/calmodulin-dependent protein phosphatase (PPP3) and is associated with the phosphorylation of GluA1 at Ser^831^ (but not Ser^845^) via PKCγ rather than PKA or CaMKII [194]. Inhibition of the PI3K/AKT pathway also prevented the phosphorylation and surface expression of AMPARs containing GluA1, alleviating pain behavior in a rat model and attenuating central sensitization, thereby relieving IS-induced chronic migraine [195]. This suggests the existence of differential phosphorylation regulatory pathways for GluA1 and GluA2 in neuropathic pain, and modulation of the membrane surface content of CP-AMPARs through different phosphorylation pathways may be crucial. Epilepsy, characterized by excessive excitation of hippocampal neurons and overactivation of AMPARs, is closely related to AMPAR phosphorylation. Vezatin, abnormally expressed in epilepsy patients, interacts with PKA. Knockdown of vezatin reduces the level of phosphorylated PKA (pPKA), thereby reducing the phosphorylation of GluA1 at the S845 site, downregulating the surface expression of the GluA1 subunit, and inhibiting seizures in epileptic mice. This suggests that the pathological mechanism of seizures may involve the regulation of GluA1 phosphorylation on seizure intensity [196].

A decrease in total AMPAR levels with higher turnover and enhanced AMPAR ubiquitination has been observed in AD patients, as well as in neurons and brain tissues exposed to Aβ. The E3 ligase Nedd4, which is involved in AMPAR ubiquitination, was found to increase in the AD brain, while the expression of the AMPAR deubiquitinase USP46 decreased. These changes in enzyme levels are part of the process through which Aβ leads to a reduction in AMPARs, suggesting that AMPAR ubiquitination is a crucial molecular event that leads to the loss of AMPARs and inhibition of synaptic transmission in AD [197]. Aβ-induced reduction of synaptic AMPARs is prevented, while the phosphorylation of GluA1 increases in neurons of mice with ubiquitin defects. Consistently, the detrimental effects of Aβ on synaptic AMPARs were also inhibited in S845-deficient phosphomimetic mutant mice, accompanied by enhanced ubiquitination of GluA1 [198]. This suggests that the pathogenesis of AD may involve cross-regulation between Aβ-induced GluA1 phosphorylation and ubiquitination.

In individuals with epilepsy, at least three *Nedd4-2* missense mutations have been identified, which disrupt GluA1 ubiquitination by reducing the interaction with the scaffolding protein [168]. Studies on mice with Nedd4-2 loss of function have shown that the loss of Nedd4-2 function impairs GluA1 ubiquitination, leading to neuronal hyperexcitability and epileptic seizures [168]. In addition, it has been found that under conditions of epileptic seizures, pyridoxal-5′-phosphatase/chronophin (PLPP/CIN) dephosphorylates the Nedd4-2 S448 site, leading to a decrease in Nedd4-2 protein levels. Loss of PLPP/CIN results in enhanced Nedd4-2-mediated ubiquitination of GluA1 subunits, reducing the responsiveness of AMPARs during epileptic seizures and interrupting the progression of seizures [176].

In addition to ubiquitination, lysine acetylation of AMPARs is also involved in the progression of AD. AMPAR acetylation is significantly reduced in AD and Aβ-exposed neurons. Enhancing GluA1 acetylation ameliorates Aβ-induced overall surface AMPAR reduction. In this process, p300 acts as the acetyltransferase responsible for AMPAR acetylation, and the expression of acetylated GluA1 mimetics has been demonstrated to rescue AD-mediated synaptic plasticity and memory impairment in APP/PS1 mice [199]. In other words, regulating AMPAR membrane stability through AMPAR acetylation may be an effective molecular target for restoring synaptic pathology and memory deficits in AD.

The dysregulation of AMPAR palmitoylation may lead to heightened neural excitation, thereby compromising the stability of neural networks and precipitating seizures. Mice lacking the Cys1 palmitoylation site of GluA1 (C811) exhibit an enlargement of LTP-induced spines and increased susceptibility to seizures, attributed to the palmitoylation defect, while re-expression of AMPARs restores normal excitatory synaptic transmission [188]. AMPAR palmitoylation is also implicated in addictive behaviors within the nervous system. Cocaine administration transiently increases the palmitoylation of GluA1 and GluA3 in the nucleus accumbens (NAc), subsequently leading to AMPAR internalization, which is associated with the reward system in addictive disorders. Pre-administration of the palmitoylation inhibitor 2-bromopalmitate (2-BP) before cocaine administration can prevent AMPAR internalization and ameliorate the subjects’ behavioral response to cocaine [200].

## 10. Discussion

In recent years, studies have gradually revealed the trafficking mechanism of AMPARs and associated synaptic functions; however, the structure of AMPAR also affects the synaptic function to some extent, and its N-terminal domain-mediated metastable gating regulation is still unclear. Based on molecular information on the interface and topology of AMPAR interactions with its auxiliary proteins, it may be possible to further elucidate the role of its N-terminal structural domain in synaptic transmission and plasticity. Furthermore, how key residues at the interaction interface transduce their effects during gating regulation also needs to be further clarified. And whether there is NTD-mediated allosteric gating in AMPAR as observed in NMDAR remains to be answered [201]. In addition to the N-terminal structural domain, the C-tail plays a key role as a recipient of phosphorylation/dephosphorylation modifications and as a docking site for many auxiliary proteins; however, up to now, there has been no systematic research on the structure of the C-tail [202].

Since AMPARs are excitatory synaptic receptors in the CNS, their expression at the synaptic surface may have important implications for human cognitive behaviors. The researchers believe that, apart from the structural aspect, the current pressing issues in the AMPARs field may include whether the diffusion and recruitment of AMPARs in vivo are induced during learning. Furthermore, is PDZ interaction absolutely necessary for the diffusion and recruitment of AMPARs? and whether AMPAR diffusion and recruitment are obligatory steps for all synaptic LTP [76]. RNA aptamers hold great promise as a treatment for AMPAR or other CNS diseases related to the ionotropic glutamate receptor family [202]. But how does miRNA activity respond to neuronal activity, especially upon AMPAR or metabotropic glutamate receptor stimulation? Beyond that, the signaling pathways that translate synaptic activity into miRNA transcriptional regulation of the AMPAR subunit or related protein expression remain unclear [203]. Synaptic plasticity is also crucial in disease treatment. Mechanisms of AMPAR trans-synaptic localization and coordinated intracellular and extracellular responses to synaptic plasticity remain unclear [204]. The question here is as follows: how do some compounds affect synaptic plasticity (LTP and LTD)? Do they affect the level of region-specific AMPAR synapses? The mechanisms by which these compounds affect cognition, memory, behavioral flexibility, anxiety, and depression-like behavior in different animal models have not been fully understood. Moreover, based on AMPAR research [205], it is also possible to develop validated biomarkers related to human neuroplasticity.

Changes in the abundance of post-synaptic AMPARs are a fundamental mechanism for most forms of synaptic plasticity [10]. We have highlighted the complexity of the AMPAR signaling apparatus, including the interplay with auxiliary subunits at multiple levels and the combined effect of PTMs. Associated proteins involved in these processes control the following properties and/or processes: surface density and distribution of AMPARs in both synaptic and extra-synaptic sites of the plasma membrane, formation and maturation of synapses, and filling of the reserve pools, which contribute to the generation of functionally diverse signaling mechanism. The main challenges for future research include a better understanding of the spatiotemporal expression patterns of AMPAR, the differential regulatory mechanisms of CI-AMPARs and CP-AMPARs, and the precise stoichiometry of each subunit. Additionally, it is necessary to focus on possible interactions between these related auxiliary proteins. In the future, further functional studies with the assistance of high-resolution microscopes will clarify how auxiliary subunits and synaptic interaction partners regulate the complex functions of AMPAR, which is very promising for clarifying the diverse information processing in the brain. This also implies that AMPARs have great potential for studying the pathogenesis of cognitive impairment, including neurodegenerative diseases, may help to compensate for the impairment of activity-dependent plasticity observed in a variety of neuro(degenerative) diseases, and may provide new ideas for the treatment of human cognitive disorders.

## Figures and Tables

**Table 1:** Functions and sites of action of AMPAR trafficking-related proteins.

	Interactor	Proposed Function	Interaction Subunits	Citations
Vesicle formation	Rab11	AMPAR is transported in Rab11-positive recycling endosomes along microtubule tracks within the dendritic shaft	GluA1	[26]
	CaMKII	Phosphorylates Stg and GluA1 as well as facilitates the localization and transport of GluA1	GluA1	[35]
Vesicle trafficking	MAP	Binds to microtubules and microfilaments, mediating or regulating the interaction between axonal microtubules and actin filaments		[43]
	Actin	Promotes receptor localization and provides transport routes and power	GluA1	[47]
Post-synaptic site of AMPAR	PSD-95	Induces AMPARs to localize on the PSD	GluA1	[54,58]
	SAP-97	Promotes the expression of GluA1 at the membrane	GluA1	[70]
	PKA	Promotes the expression of GluA1	GluA1	[78]
	PKC	Reduces the affinity of GRIP1/2 for GluA2 and promotes the internalization of GluA2	GluA2	[97]
	EPB41L1	AMPAR is anchored to the PSD by SAP97 and EPB41L1	GluA1	[100]
Vesicle fusion	SNARE complex	Mediates vesicle localization and membrane fusion	GluA2	[106]
	NSF	Promotes the decomposition of SNARE complexes to accelerate circulation and increase the expression of AMPARs at the membrane	GluA2	[110]
	AP-2	Induces the internalization of AMPARs	GluA2	[116]

**Table 2:** Neurological diseases and functional sites of AMPAR PTMs.

AMPAR PTM	Functional Subunits and Sites	Related Proteins	Diseases	Citations
Phosphorylation	GluA2 (S880)	/	AD	[192]
	GluA2 (S880)	PKCα, PICK1	Neuropathic Pain	[152,193]
	GluA1 (S831)	PKC γ, PI3K/AKT	Neuropathic Pain	[194,195]
	GluA1 (S845)	Vezatin, PKA	Epilepsy	[196]
Ubiquitination	GluA1 (S845)	Nedd4, USP46	AD	[197,198]
	GluA1 (S845)	Nedd4-2, PLPP/CIN	Epilepsy	[168,176]
Palmitoylation	GluA1(K813, K819, K822, K868)	P300	AD	[199]
Acetylation	GluA1 (C811)	Cys1	Epilepsy	[188]
	GluA1, GluA3	/	Addictive Disorders	[200]

## Abbreviations

AMAPR, α-amino-3-hydroxy-5-methyl-4-isoxazoliproic acid receptor; NMDAR, N-methyl-D-aspartate receptor; LTP, long-term potentiation; LTD, long-term depression; AD, Alzheimer’s disease; ID/IDD, intellectual disability/intellectual developmental disorder; ASD, autism spectrum disorder; SCZ, schizophrenia; ER, endoplasmic reticulum; GA, Golgi apparatus; CT, cytoplasmic tail; PSD, postsynaptic density; PSD-95, postsynaptic density protein 95; SAP-90, synapse-associated protein 90; PSD-93, postsynaptic density protein 93; SAP-97, synapse-associated protein 97; SAP-102, synapse-associated protein 102; MAGUK, membrane-associated guanylate kinase; PDZ, PSD-95/Dlg/ZO-1; SH3, Src homology 3; GK, guanylate kinase; Stg, stargazin; EPSC, excitatory postsynaptic current; EPB41L1, erythrocyte membrane protein band 4.1-like 1; NSF, N-ethyl maleimide-sensitive factor; SNAP, soluble N-ethyl maleimide-sensitive factor attachment protein; SNARE, soluble N-ethyl maleimide-sensitive factor attachment protein receptor; v-SNARE, vesicle SNARE protein; t-SNARE, target SNARE protein; Syb-2, synaptobrevin-2; Stx-3, synaptin-3; Syt-1, Synaptotagmin-1; AP-2, adaptor protein 2; PKA, protein kinase A; CP-AMPAR, calcium-permeable AMPAR; PKC, protein kinase C; GRIP1, glutamate receptor-interacting protein 1; PICK1, protein interacting with C kinase 1.
